# Recurrence hyperparathyroidism caused by synchronous parathyroid carcinoma and parathyromatosis in a patient with long-term hemodialysis

**DOI:** 10.1186/s12882-023-03328-6

**Published:** 2023-10-04

**Authors:** Jun Yang, Xili Lu, Pingping Zhou, Hao Liu, Jili Wang, Xinhui Su

**Affiliations:** 1 Department of Nuclear Medicine, the First Affiliated Hospital, College of Medicine, Zhejiang University, Hangzhou, 310003 P.R. China; 2 The Department of Pathology, the First Affiliated Hospital, College of Medicine, Zhejiang University, Hangzhou, 310003 P.R. China

**Keywords:** Recurrence hyperparathyroidism, Parathyroid carcinoma, Parathyromatosis, Hemodialysis

## Abstract

**Background:**

Parathyroid carcinoma and parathyromatosis are very rare diseases in patients on hemodialysis. Its pathogenesis, clinical features, preoperative diagnosis, and surgery are challenging. We describe a rare case of recurrent hyperparathyroidism due to synchronous parathyroid carcinoma and parathyromatosis.

**Case presentation:**

A 46-year-old Chinese woman was diagnosed with end-stage renal disease and received regular hemodialysis. Four years later, she experienced discomfort due to itching and was diagnosed with drug-resistant secondary hyperparathyroidism. Parathyroidectomy was performed, and her parathyroid hormone (PTH) levels were reduced. The pathology also revealed that the four nodules were parathyroid nodular hyperplasia without evidence of malignancy. Five years after surgery, the right subcutaneous nodule and left inferior nodule were detected by multiple imaging modalities, and the nodules were accompanied by recurrence itching and elevation of PHT. A complete resection of two nodules was performed, and the patient was diagnosed with parathyroid carcinoma and parathyromatosis. At 8 months postsurgery, her PHT and serum calcium levels were stable, and there were no signs of recurrence.

**Conclusions:**

This is a rare case of synchronous parathyroid carcinoma and parathyromatosis in a patient with secondary hyperparathyroidism after parathyroidectomy. We suggest meticulous handling of parathyroid hyperplasia to avoid rupture and spillage during surgery, and precise pro-operation location by multiple imaging modalities is crucial for successful parathyroidectomy.

## Background

Secondary hyperparathyroidism (SHPT) is an almost universal phenomenon of end-stage renal disease (ESRD) [1]. SHPT is initially a physiologic adaptation but becomes pathologic with progressive ESRD, resulting in hypercalcemia, osteodystrophy and cardiovascular morbidity, in addition to other manifestations [2, 3]. SHPT is initially treated with medical management but often necessitates parathyroidectomy for definitive treatment when pharmacologic treatment is not effective [1, 4]. Removal of all hyperplastic parathyroid glands is crucial for parathyroidectomy, as remnant parathyroid tissue can be persistently stimulated from ESRD, leading to recurrence or persistent SHPT, which occurs in 2.0-9.2% of cases [5]. In most cases, recurrence or persistent SHPT is ectopic, supernumerary glands, remnant parathyroid tissue or hyperplasia of the gland tissue autoimplanted and, very rarely, parathyromatosis or parathyroid carcinoma [4, 6–12].

Parathyroid carcinoma is a rare disease commonly caused by primary hyperparathyroidism and very rarely caused by long-term chronic stimulation due to SHPT [13, 14]. Parathyromatosis is also rare, mainly because of spillage and seeding of the parathyroid tissue during parathyroid surgery [7, 11, 12]. Preoperative clinical diagnosis, differential histopathological diagnoses between parathyroid carcinoma and parathyromatosis and subsequent treatment are challenging. We report a rare case of recurrent SHPT after parathyroidectomy due to synchronous parathyroid carcinoma and parathyromatosis in a patient with hemodialysis.

## Case presentation

**Fig. 1:**
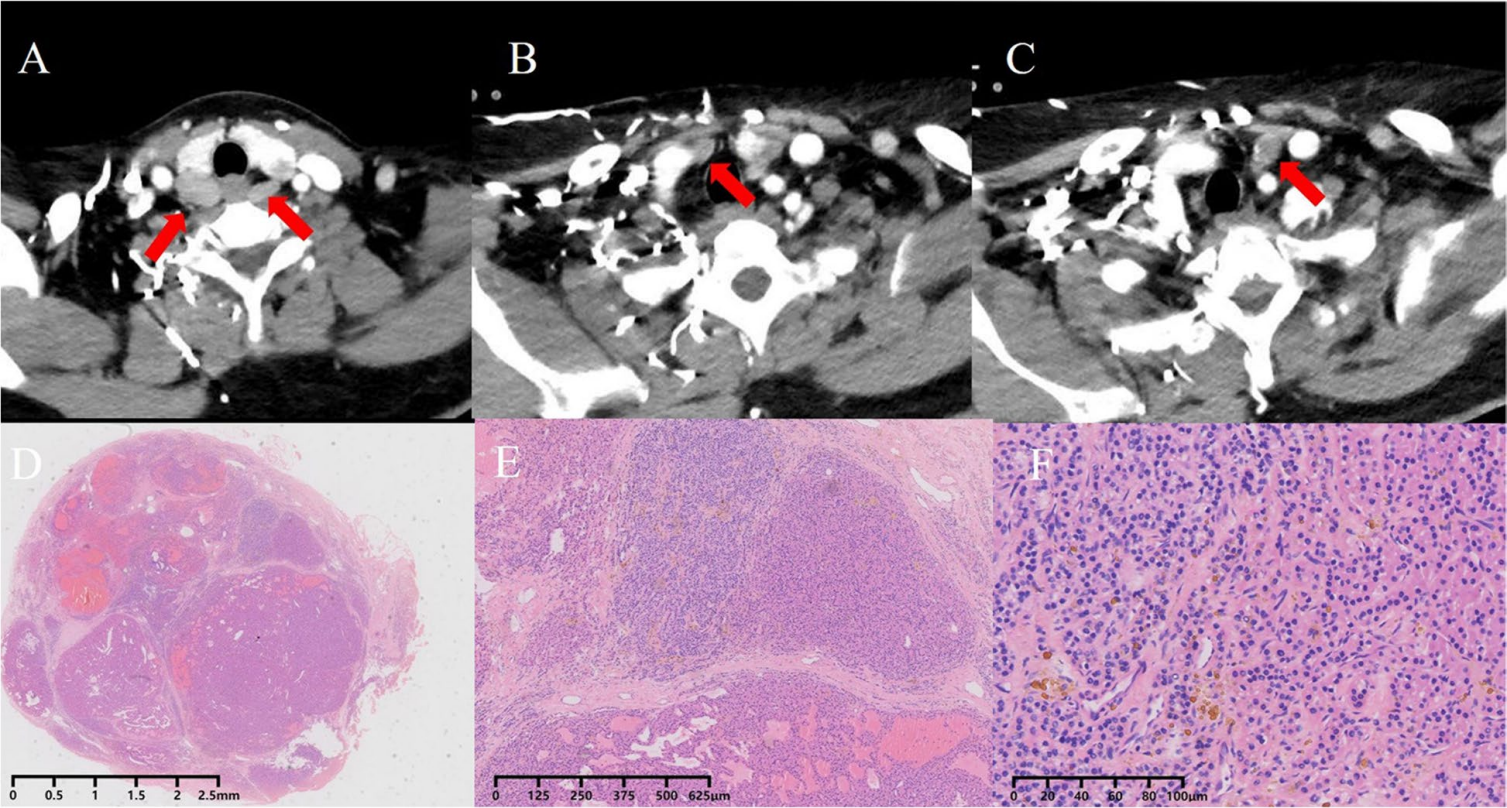
Axial arterial-phase contrast-enhanced CT scan (**A**-**C**) shows four mildly enhanced orthotopic parathyroid nodules (arrows) located posterior and inferior to the thyroid gland. Histopathology (hematoxylin-eosin staining (HE): **A** magnification ×40, **B** magnification ×100, **C** magnification ×200) revealed nodular hyperplasia composed of chief cells

A 46-year-old Chinese woman had end-stage renal disease secondary to chronic glomerulonephritis and was treated with regular hemodialysis since May 2013. During follow-up through a local hospital, the patient presented with normal serum total calcium levels, hyperphosphatemia and elevated parathyroid hormone (PTH). She was diagnosed with secondary hyperparathyroidism in August 2015. A vitamin D analog (calcitriol and caltrate) and lanthanum carbonate were recommended, but the treatment was ineffective. In October 2017, she was referred to our hospital because she constantly experienced generalized itching over the prior 1 year. Her blood test results were as follows: serum PTH 1672 pg/mL (normal 10–65); serum phosphate, 2.03 mmol/L (normal 0.87–1.45); serum calcium, 2.68 mmol/L (normal 2.03–2.54); and serum alkaline phosphatase 112 U/L (normal 40–150). A cervical contrast-enhanced computer tomography (CT) scan showed four enhanced nodules consistent with orthotopic parathyroid glands (Fig. 1). Ultrasonography also revealed four hypoechoic nodules at the posterior of the thyroid and normal echogenicity in the thyroid. No evidence was indicated that there were ectopic parathyroid glands. Parathyroidectomy with right forearm autotransplantation (from the right inferior parathyroid gland) was performed where the surgeon claimed to remove four hyperplastic parathyroid glands intact without any spillage. The final pathology report showed parathyroid nodular hyperplasia without evidence of malignancy (Fig. 1). On the first postoperative day, tests showed PTH of 74.4 pg/mL, serum calcium of 2.11 mmol/L, and serum phosphate of 1.71 mmol/L, with gradual alleviation of the itching.

**Fig. 2:**
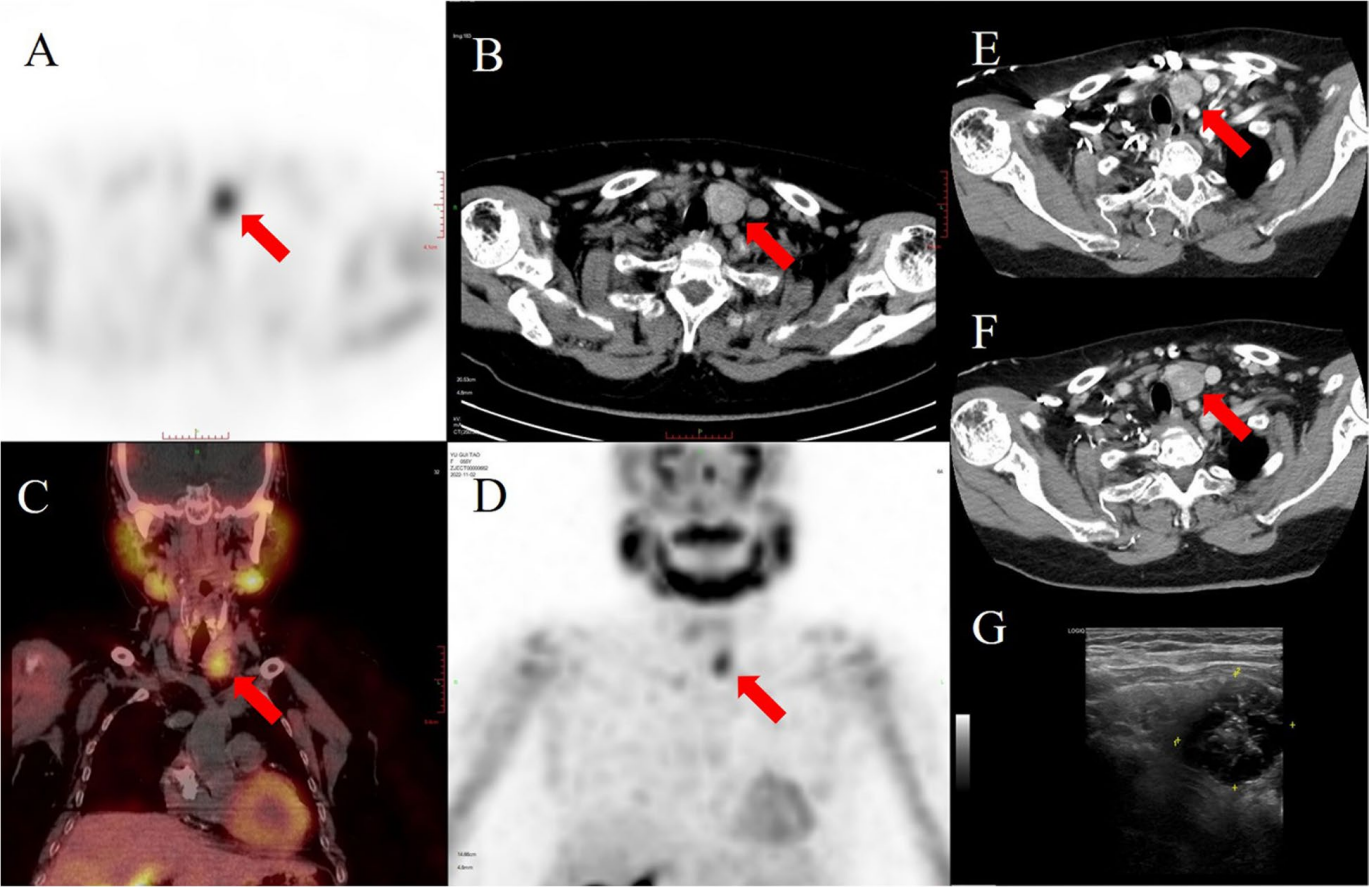
The 99mTc-MIBI SPECT/CT scan shows focused tracer accumulation with a low-density nodule located inferior to the left thyroid gland (red arrows: **A** SPECT, **B** axial image, **C** coronal SPECT/CT fusion image, **D** coronal SPECT image). Contrast-enhanced CT scan reveals arterial-phase (**D**) and delayed-phase (**E**) axial images of a moderately enhanced parathyroid nodule (red arrows) located inferior to the left thyroid gland. Ultrasonographic image of a hypoechoic nodule with irregular margins and calcareous composition also in the left inferior of the thyroid gland (**G**)

**Fig. 3:**
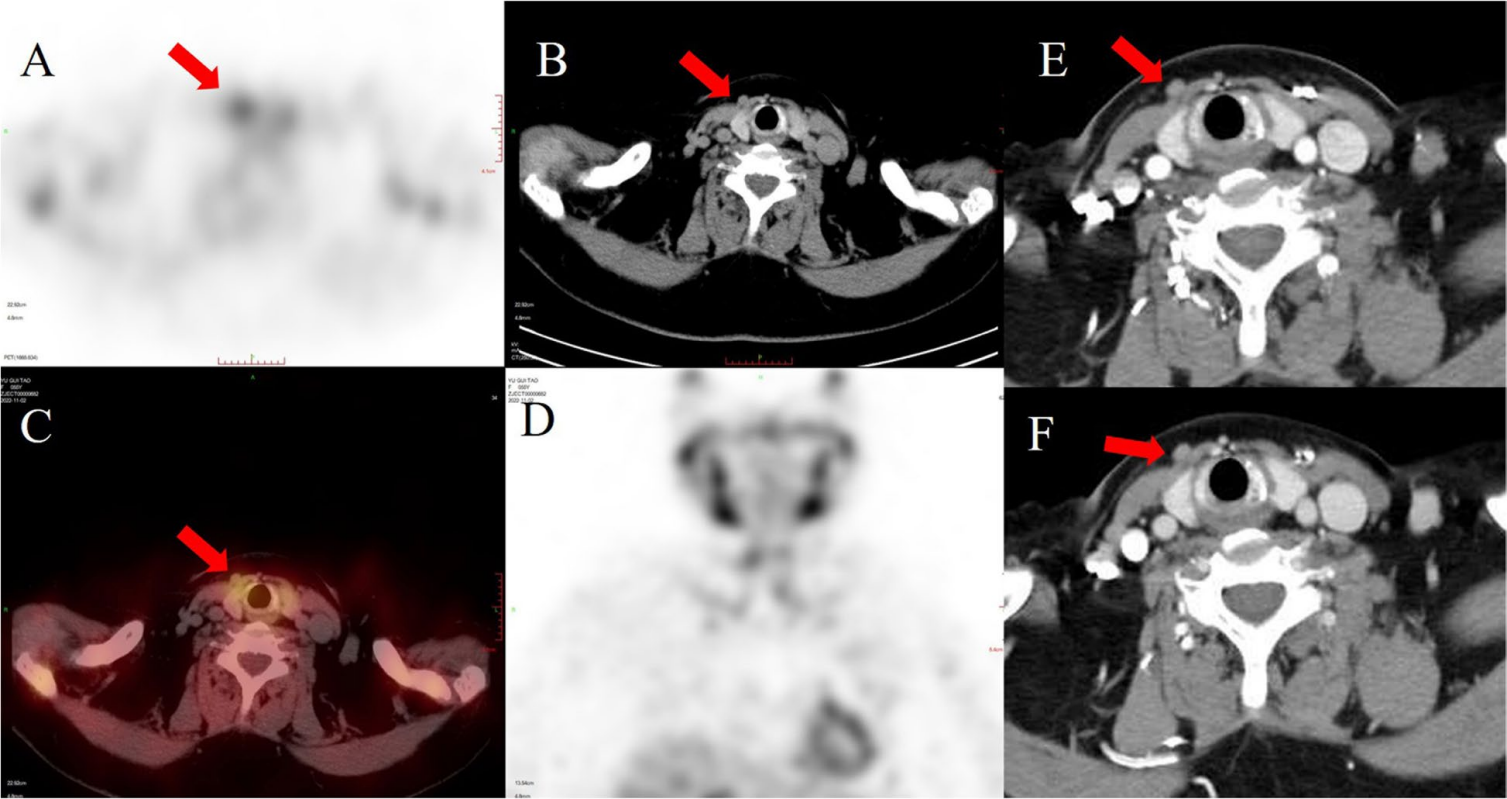
The 99mTc-MIBI SPECT/CT scan also shows a mild focus tracer accumulation with a low-density nodule located subcutaneously in the anterior neck (red arrows: **A** SPECT, **B** axial image, **C** axial SPECT/CT fusion image, **D** coronal SPECT image). Contrast-enhanced CT scan reveals arterial-phase (**D**) and delayed-phase (**E**) axial images of mildly enhanced nodules (red arrows) located subcutaneously in the anterior neck

**Fig. 4:**
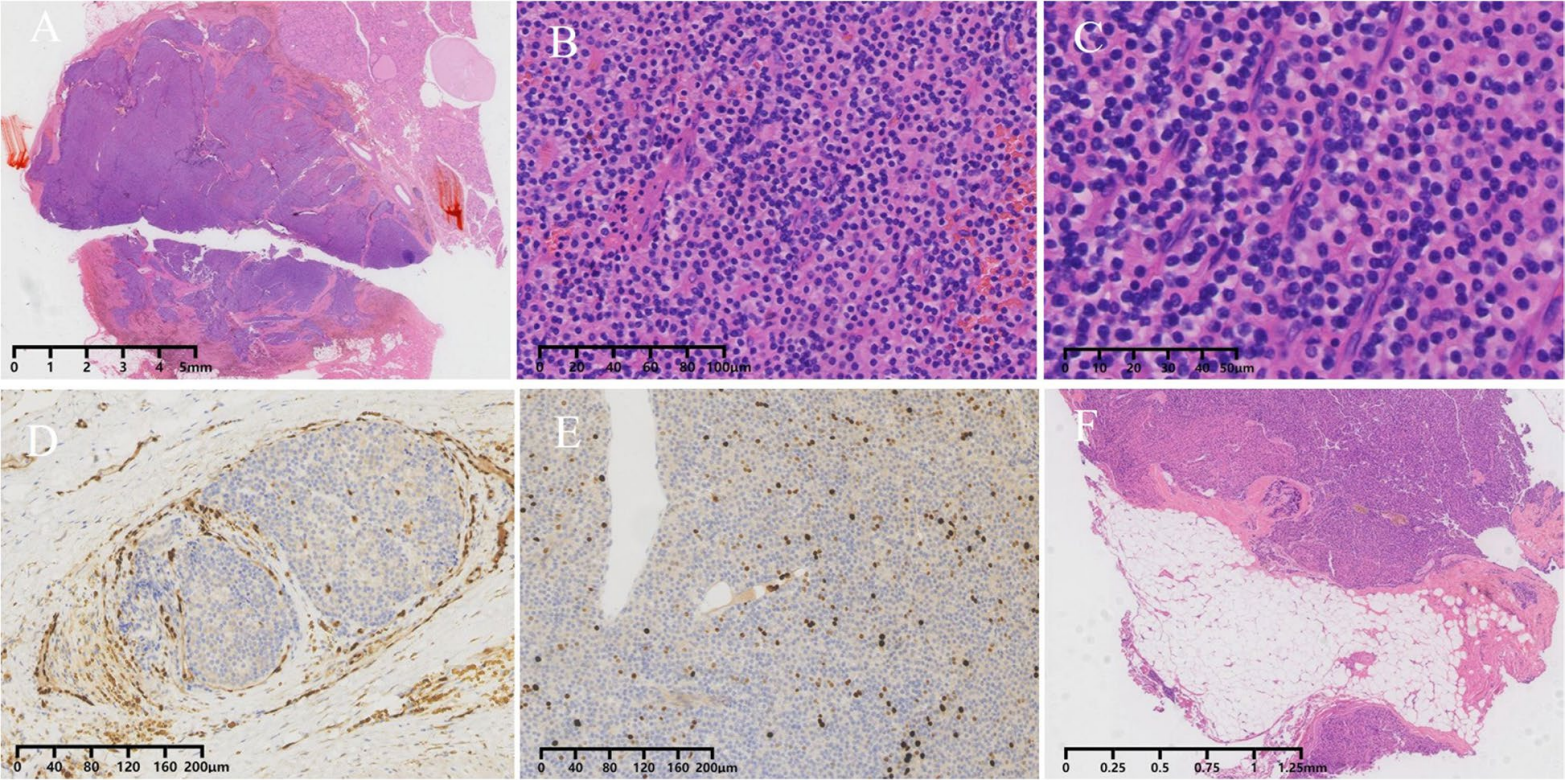
The histopathological images show parathyroid carcinoma in the left inferior of the left thyroid, which consisted of dense growth of chief cells with a fibrous capsule covering the nodule (**A** HE staining, magnification ×10). HE staining reveals solid growth of chief cells with nuclear pleomorphism and an increased mitotic count (**B** magnification ×200, **C** magnification ×400). The evidence of vascular invasion is indicated by immunohistochemical staining for ERG (**D** magnification ×100). The Ki-67 proliferation index in parathyroid carcinoma exceeded 5% (**E** magnification ×100). The histopathology of the subcutaneous nodule in the right neck shows scattering of well-circumscribed benign hyperplasia parathyroid tissue in the nodule (**F** HE, magnification ×20)

Fifteen months after the surgery, her test revealed that serum calcium and PTH levels were 2.54 mmol/L and 154 pg/mL, respectively. During this time, she presented without itching and bone pain. However, five years after surgery, she presented with back pain and gradually elevated PTH and calcium levels. On admission, PTH, serum calcium and phosphate were 3356 pg/mL, 3.22 mmol/L and 1.39 mmol/L, respectively. 99mTc-sestamibi (99mTc-MIBI) single-photon emission computed tomography/computed tomography (SPECT/CT) revealed accumulation at the inferior pole of the left thyroid gland, consistent with the findings of contrast-enhanced CT and ultrasonography (Fig. 2). A right nodule with tracer accumulation was located subcutaneously in the anterior neck, which was also identified by CT (Fig. 3). Neck exploration was performed in December 2022, and the right subcutaneous nodule (1.0 × 1.0 cm) and left inferior nodule (2.5 × 2.5 cm) with irregular margins and calcareous composition were identified and removed. On the first day after the operation, tests showed PTH of 63.5 pg/mL, serum calcium of 1.91 mmol/L, and serum phosphate of 1.30 mmol/L, with gradual alleviation of the back pain. Microscopic pathology analysis noted that the left inferior nodule consisted of dense growth of chief cells with nuclear pleomorphism and an increased mitotic count and a fibrous capsule covering the nodule. ERG staining revealed venous infiltration in some areas, and the Ki-67 proliferation index exceeded 5%, suggesting parathyroid carcinoma (Fig. 4). The subcutaneous nodule exhibited nodular proliferation of chief cells (Fig. 4).

According to the clinical presentation and pathology, parathyromatosis and parathyroid carcinoma were confirmed. During 8 months of follow-up, her back pain improved. Her PTH and calcium levels fluctuated between 190 and 320 pg/mL and 2.23–2.48 mmol/L, respectively, under regular hemodialysis.

## Discussion and conclusions

Synchronous parathyroid carcinoma and parathyromatosis are very rare causes of recurrence SHPT after parathyroidectomy in patients undergoing hemodialysis. The patient in our study underwent parathyroidectomy, and subsequent histological findings confirmed all four parathyroid lesions to be parathyroid hyperplasia. Five years later, recurrence of SHPT was diagnosed, and synchronous parathyroid carcinoma and parathyromatosis were confirmed by histopathology.

Parathyroid carcinoma is uncommon and occurs in 0.3-5% of all cases of primary hyperparathyroidism [13, 15, 16]. The etiology of this tumor is unknown. Parathyroid carcinoma is commonly sporadic as a potentially hereditary disease, which is associated with hyperparathyroidism-jaw tumor syndrome or familial hyperparathyroidism due to a pathogenic germline CDC73 variant that encodes parafibromin [14, 17, 18]. An increased incidence of parathyroid carcinoma has been associated with ESRD [19]. According to the literature, the mean age is 55 years, with approximately equal sex distribution [9]. The biochemical and clinical characteristics are characterized by markedly elevated serum calcium, PHT and target-organ damage [20]. The diagnosis of parathyroid carcinoma is also made after surgery. The histological criteria for parathyroid carcinoma are sometimes challenging, as longstanding SHPT may be associated with enlarged parathyroid glands with some atypical features, including contour irregularity that can mimic invasive growth [14, 21]. The diagnosis of parathyroid carcinoma should be considered if there is unequivocal angioinvasion, lymphatic invasion, perineural invasion or invasion into adjacent anatomic structures [14]. In our case, the invasion of blood vessels was definite, highlighted by immunohistochemical staining for ERG. Multiple full-thickness capsules were also observed. However, invasion into adjacent structures was absent, and the mitotic features of parathyroid cells were remarkable. Some reports claim that a benign hyperplastic nodule may be converted to a carcinoma by a prolonged hypocalcemic stimulus in patients with hemodialysis and that PTH is produced largely by malignant cells [22].

Parathyromatosis is another uncommon and challenging cause of SHPT recurrence. Pathology of parathyromatosis always reveals multiple nests of hyperplastic parathyroid with neck or mediastinum and lack of a real capsule [21–23]. The exact etiology is still unknown, and two theories have been accepted. The first hypothesis (type 1) is that parathyromatosis may be the result of the overgrowth of preexisting parathyroid nests of embryological origin under the influence of physiological stimuli such as ESRD. The second hypothesis (type 2) is that parathyroid tissue spills and seeds inside the operative field during surgery [23]. Our case is supported by type 2 because the parathyromatosis was located subcutaneously in the right anterior neck, which was the operative field, and the nodule grew progressively after parathyroid surgery. The histopathology was also confirmed to be parathyromatosis without evidence of malignancy of the parathyroid gland. Although the first operative report on the four nodular hyperplasia parathyroid nodules removed makes no mention of rupture or spilling of parathyroid tissue, it can still be a possible cause of this parathyromatosis.

The only curative treatment for parathyroid carcinoma and parathyromatosis is surgery, and the best chance of cure can be acquired with en bloc excision at the first operation [20]. During surgery, it is crucial to minimally manipulate and avoid rupture of the capsule and spill over of the tumoral cells in the operative field, especially in patients with hemodialysis. In our case, rupture and spilling of parathyroid tissue may have occurred, and the existing factor of metabolic derangements in ESRD persistently stimulated parathyroid hyperplasia. Synchronous parathyroid carcinoma and parathyromatosis occur in the same patient, and we speculate that different stages of parathyroid hyperplasia, even when converted to carcinoma, exist due to the heterogeneity of parathyroid tissue and different expression levels of calcium-sensing receptors under the same stimuli [7, 22, 24]. Precise location of all small parathyromatosis and diagnosis of parathyroid carcinoma is difficult, but it enhances the chances of removing all culprit nodules [25]. The first-line localization procedure includes ultrasonography, 99mTc-MIBI SPECT/CT and enhanced-contrast CT, which have various advantages and disadvantages [26]. Local expertise, surgeon preference and the patient’s clinical scenario are important considerations when determining the type of localization modality. A multimodality approach is ultimately desirable, particularly in challenging conditions such as multigland disease, persistent or recurrent SHPT or parathyromatosis. Fortunately, two nodules were revealed by enhanced-contrast CT and 99 m Tc-MIBI SPECT/CT in our case, but only 40% of patients have been successfully diagnosed preoperatively [26, 27].

Parathyroid carcinoma and parathyromatosis recurrence occur in more than 50% of patients during follow-up, and locoregional recurrence and/or metastases usually occur 2–3 years after surgery [28, 29]. The prognosis of parathyroid carcinoma is unfavorable. According to the literature, the 5-year and 10-year survival rates are 85–91% and 49-87.6%, respectively [28–30]. Regarding our case, at 8 months of follow-up, she was alive and had stable PTH and calcium levels. If surgical intervention fails, calcium mimetic, bisphosphonate or radioactive therapy should be considered.

In conclusion, synchronous parathyroid carcinoma and parathyromatosis in a patient with hemodialysis are very rare causes of recurrence SHPH. Meticulous handling of parathyroid hyperplasia to avoid rupture and spillage during surgery and precise pro-operation location by multiple imaging modalities are crucial for successful parathyroidectomy.

## Data Availability

Not applicable.

## Abbreviations

SHPT: Secondary hyperparathyroidism
ESRD: end-stage renal disease
PTH: parathyroid hormone
CT: computer tomography
^99m^Tc-MIBI: ^99m^Tc-sestamibi
SPECT: single-photon emission computed tomography/computed tomography
